# Challenges and opportunities of power systems from smart homes to super-grids

**DOI:** 10.1007/s13280-015-0733-x

**Published:** 2015-12-14

**Authors:** Philipp Kuhn, Matthias Huber, Johannes Dorfner, Thomas Hamacher

**Affiliations:** Institute for Renewable and Sustainable Energy Systems, Technische Universität München, Munich, Germany

**Keywords:** Renewable energy, Sustainability, Scaling, Transformation of energy systems, Socio-technical complexity

## Abstract

The world’s power systems are facing a structural change including liberalization of markets and integration of renewable energy sources. This paper describes the challenges that lie ahead in this process and points out avenues for overcoming different problems at different scopes, ranging from individual homes to international super-grids. We apply energy system models at those different scopes and find a trade-off between technical and social complexity. Small-scale systems would require technological breakthroughs, especially for storage, but individual agents can and do already start to build and operate such systems. In contrast, large-scale systems could potentially be more efficient from a techno-economic point of view. However, new political frameworks are required that enable long-term cooperation among sovereign entities through mutual trust. Which scope first achieves its breakthrough is not clear yet.

## Introduction to the challenges ahead

The integration of intermittent energy sources in the power system creates manifold challenges and problems, which have not been overcome yet. Breakthroughs in system design, transport and storage technologies, as well as economic organization are necessary.

The distributed nature of renewable generation contradicts the traditional power system structure with large centralized plants. This contradiction opens the discussion whether a new fundamental design is necessary. On the one hand, decentralized generation suggests shifting to small-scale systems, whereas the intermittent nature of generation favors large-scale systems. To facilitate discussion, we categorize the various approaches in three scopes, which roughly refer the geographical scale: local, national, international.

Local scope: the local scope reaches from individual homes to cities. The discussion is focused on politically or privately motivated plans to become energy autonomous. Furthermore, decentralized technologies like photovoltaic (PV) have reached grid parity which allows for cost reductions through own production.

National scope: at the national scope, governments can set the agenda for energy policy and regulation. The current setting of these conditions is a result of political decisions in the past (e.g., the Renewable Energy Sources Act in Germany) and national objectives for the future (e.g., increase share of renewables, meet greenhouse gas emission targets).

International scope: the discussion at this scope is led by the awareness that a larger system reduces the integration challenge due to smoothing effects from variable generation of wind and solar. Furthermore, system planning at this scope enables the participation of larger areas with a high potential in a common European market (e.g., high PV-potential in southern countries; high wind potential in northern countries; large storage potential, e.g., in Norway).

In all three scopes, the integration of fluctuating renewables is being realized currently. Which scope(s) are to be preferred when trying to develop towards a more sustainable energy supply is a point of active discussion. By themselves, visions on each scope are reasonable.

A widely accepted new fundamental power system design does not exist yet. Current discussion is largely limited to individual proposals and demonstrations of individual solutions. The question is then what the general solution will look like: top-down or bottom-up? The top-down solution would create a new system design as a result of new regulation following a pre-defined master plan. In a bottom-up solution, a new system would emerge from individual projects. Regulation in that case would adapt to new issues as they occur. The path which will finally be chosen is not obvious at the moment. What became quite obvious in recent years is that decisions of individual countries, like the strong growth of intermittent, renewable energy sources in Germany, are not without impact on the neighboring countries. This leads to the observation that decisions on any scope cannot be made independently from one’s surroundings.

This paper shows and discusses results of energy models which are developed and used at the Institute for Renewable and Sustainable Energy Systems at Technische Universität München. The mentioned scopes are addressed by multiple approaches.

Challenges and opportunities of different system granularities are discussed and brought into a theoretical framework. As a conclusion, we identify a trade-off between technological and political challenges depending on the investigated scope and system sizes.

## Local solution

### Motivation/intended effect

In recent discussions about the future electricity supply, the phrase of energy autonomy came high to the agenda. The idea of very local energy and electricity systems can be approached from the perspective of smart homes, where individuals want to be independent of grid supply by generating electricity with PV and combined heat and power (CHP) plants, up to the scale of cities and political regions that claim to develop plans for getting independent of external energy supply.

### Short literature review

The literature on this topic is broad and we focus on several aspects here that analyze the techno-economic possibilities for renewable integration in decentralized electricity systems.

There is research in the direction of energy autonomous homes that tries to figure out the maximal degree of autonomy that can be reached by decentralized supply. Much of the research in the field focusses on the German market as feed-in tariffs triggered PV installations on many homes and people are now thinking about new business models including increased self-supply. Staudacher (2012a) investigated whether autonomy could be economically beneficial and found that there is no chance for profitability in the current system with current price structures. In Staudacher (2012b), the same authors show that the PV/Battery systems would allow a degree of autonomy of 50–60 %, but values higher than that can hardly be achieved. Quaschning (2012) found that even though fully autonomous electricity supply will not be economical, the addition of PV systems in homes can be very helpful in reducing the electricity bill. The employment of electric heaters helps utilize excess electricity and improves profitability.

Increasing the system size leads us from individual homes to small microgrids. Marnay and Venkataramanan (2006) show several advantages of microgrids that include the chance for combined heat and power plants (CHP) to be employed efficiently, increasing reliability of supply, and a centralized planning of the entire grid. Hatziargyriou et al. (2007) give an overview of past and ongoing research in the field around the globe. They find differing foci and drivers for research and applications in different places. Whereas in Europe environmental benefits are most important, other regions are researching the topic because of reliability reasons. Our research has to be seen in the European context with a focus on chances for integration of renewables and the chances to get independent of electricity delivery from utilities.

The study of energy autonomy in cities is part of the broader study of urban metabolism, last broadly reviewed in Kennedy et al. (2011) and recently extended by Pincetl et al. (2012). The idea is to focus on a city’s input and output flows of goods and energy. The ultimate goal is to develop closed cycles for each major flow, resulting in a sustainable operation of the whole system. Lund (2011) investigates techno-economic aspect of satisfying significant parts of urban energy demand using a spatial–temporal model and case studies for the two cities Helsinki and Shanghai. He finds that power-to-heat technologies, together with sufficiently sized local thermal storage could greatly contribute to higher RES integration on the local scale.

### Own research: Model/scenario description

In order to show the challenges that lie ahead for decentralized electricity systems, we show scenario results researching the level of autonomy that can be reached by PV systems in individual homes, in small communities, as well as in cities. Two different models are applied for the research presented here.

The first model that is applied to individual homes and small communities is a linear optimization-based dispatch model that finds a cost-minimal solution for the dispatch of storage, electricity from grids, and CHP power plants. Real-world data for 15-min electricity load were available. Furthermore, generation time series for PV plants was used as developed by Janker (2015). A description of the model and the data applied can be found in Huber et al. (2013a).

The second model operates on the district to urban scale. It is an optimal planning model for urban district heating networks. Given are locations and prices for heat generation, as well as peak and annual data for heat consumption per building. Demand data are aggregated to up to 10^4^ street segments to arrive at a manageable mixed integer linear problem. A detailed mathematical description and case study are given in Dorfner and Hamacher (2014).

### Own research: Findings

**Fig. 1:**
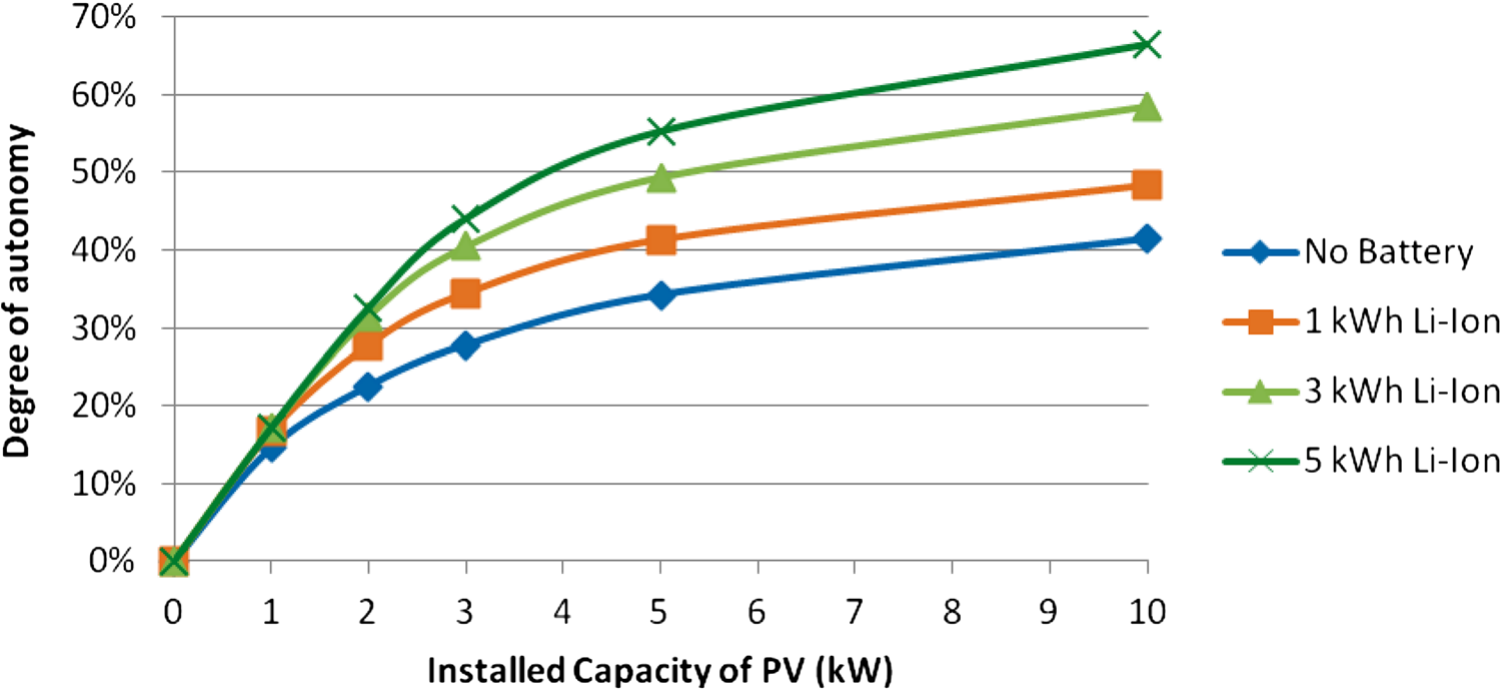
Degree of autonomy for homes with PV systems and batteries of different sizes (Huber et al. 2013a)

One central question to be answered is the maximal degree of autonomy that consumers can reach by installing decentralized generation like PV and CHP as well as local storage. Figure 1 shows the degree of autonomy plotted over the installed capacity of PV for a home in Bavaria. The blue line illustrates the case without storage option. The degree of autonomy will be less than 50 % even with 10 kW of installed PV capacity. Adding battery storage helps integrate larger shares of the produced electricity but, as the graph shows, the potentials are still limited. A battery of 5 kWh which can be considered large for a home application increases the degree of autonomy from 42 to 65 % for the largest considered PV capacity. However, a fully autonomous system would require tremendously high storage and PV capacities that are not economically viable in the near future.

**Fig. 2:**
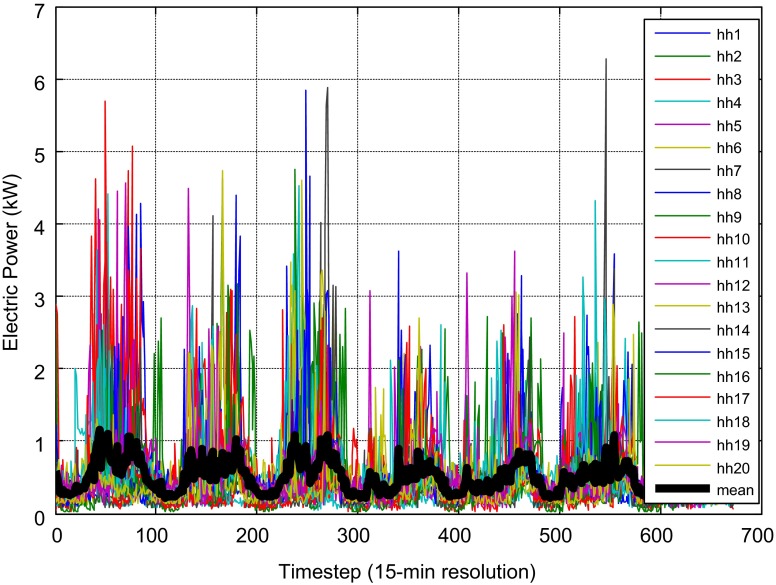
Flattening of load through connecting multiple homes in a microgrid (Huber et al. 2013b). The figure shows the individual load (colored lines) of 20 neighboring homes in a small community as well as their average load (*black line*)

One possibility to increase the chances for an efficient decentralized renewable supply might lie in the connection of several homes to a microgrid. There are several reasons supporting that idea which include economics and reliability. Economic advantages stem from the possibility to employ larger plants and from the balancing of load as depicted in Fig. 2. A clear flattening is observed and peaks are reduced dramatically in the microgrid. It seems obvious that it is easier to supply the average load (black curve) than the highly volatile loads. Higher degrees of autonomy at lower costs can be expected for those microgrids compared to individual homes.

**Fig. 3:**
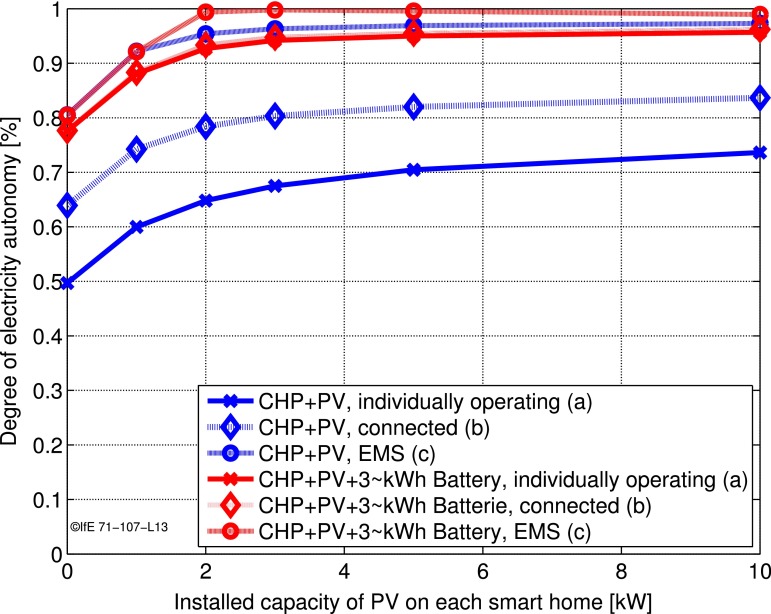
Degree of autonomy for a community which is either operating individually, connected, or even connected and managed by an energy management system (EMS) (Huber et al. 2013b)

Figure 3 shows the reached degree of autonomy for this microgrid including PV, battery, as well as CHP plants. The blue curve shows the case without storage and indicates very positive effects of a microgrid. The degree of autonomy raises from 65 to 95 % for a system with 5 kW PV and a CHP plant when the homes get connected and managed centrally. The study shows that microgrids are especially beneficial when small-scale CHP units are used and when batteries are not employed.

**Fig. 4:**
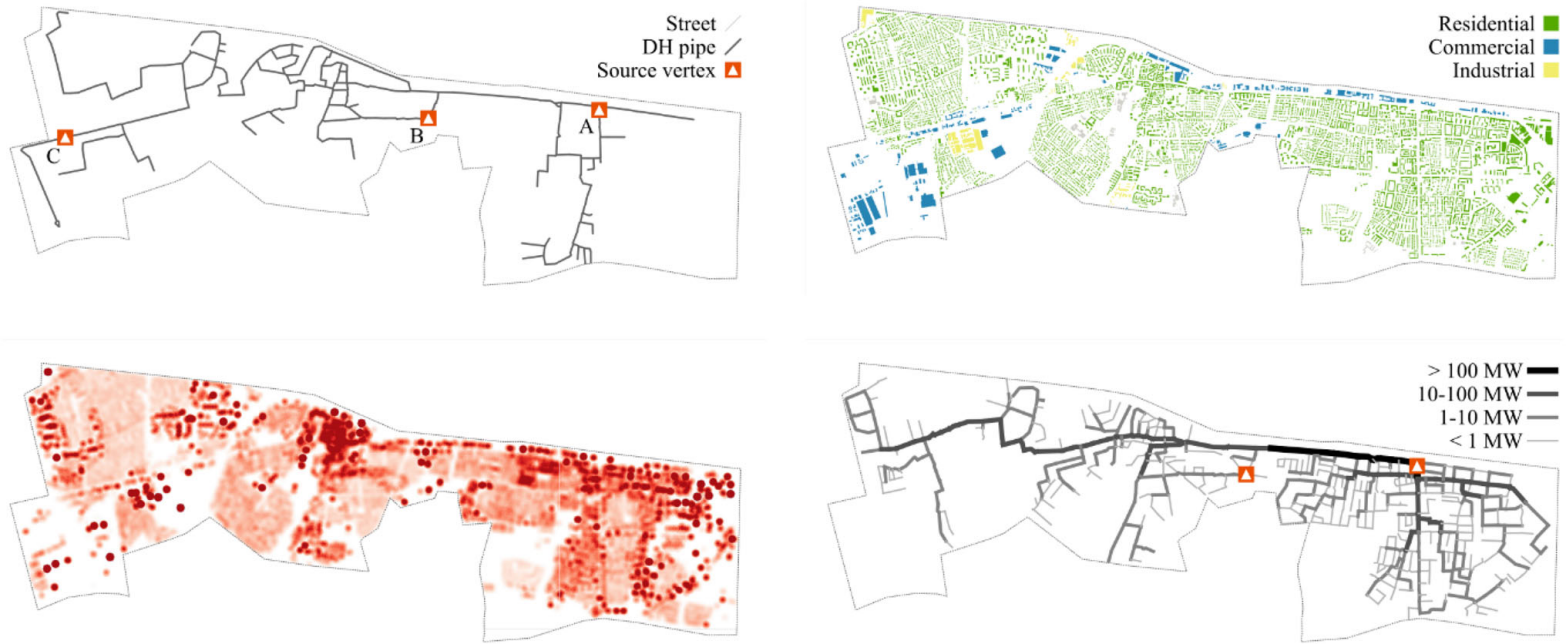
District heating expansion planning in Munich: reference plan (*top left*) and optimization result (*bottom right*), derived from building stock (*top right*) and its estimated heat demand (*bottom left*) (Dorfner and Hamacher 2014)

The case of district heating networks in cities can serve as a historical reminder for the benefits of cooperation among neighbors. Figure 4 shows the results of a case study conducted for a part of Munich (Dorfner and Hamacher 2014). In the bottom right, the figure shows the cost-optimal extension of a district heating network, given an estimated heat demand. The model automatically determines structure and size of the most economic network. In this case study, the mathematical optimization yields a similar macro-structure as a published grid expansion plan (top left). The economic feasibility of local heat distribution comes from the higher efficiency of large-scale heat generation in CHP plants. Another benefit of such a network is that only a small number of heat generators must be improved to meet new emission or efficiency targets. This can give municipal utilities more options to follow future regulations. Efficiency and flexibility of district heating could make a strong case for extending its use in the future, especially with new sustainable sources for heat energy emerging.

### Future: Chances and regulatory problems

A fully renewable-based system seems hard to be achieved at this local level. However, there will be several advantages of the small-scale systems that should lead to research efforts in several directions: Small-scale microgrids seem to be a promising approach for increasing the autonomy and the security of supply. Microgrids will require new control architectures requesting research into that direction. Another highly important technology that requires research is decentralized storage. Therefore, one major goal of research on storage should lie in trying to achieve cost reductions. The chances for the integration of renewable energies at the local level are limited and fully renewable systems seem difficult to be achieved even at the level of cities. Power-to-heat technologies, together with thermal storage, can increase the achievable integration of local RES shares. While technologies set limits for arbitrarily high shares of renewables at this level, the regulatory framework and individual incentives are already allowing to increase the utilization of solar PV and decentralized storage. The major challenges are thus technology driven and generating electricity at local level will possibly lead to increased costs of supply.

## National solution

The country scope regards the integration of the increasing amount of fluctuating renewables as a national challenge. One technical integration measure, which can be established independently without international agreements, is large-scale electricity storage.

### Motivation/intended effect

Intermittent power sources show variations on various time scales with the diurnal and the seasonal variations being the most obvious ones, but also weather phenomena lead to variations of a couple of days (Heide et al. 2011). Storage capacities need to reflect these variations. Pumped hydro, compressed air, or new thermal storage options offer storage capacities for a couple of hours, allowing diurnal storage. Seasonal storage can only be achieved by very large pumped hydro plants or chemical storage options like hydrogen. Decentralized options like batteries and super-capacitors turned out to be too expensive in the past (German Energy Agency 2010). New developments that leverage new technologies or simply economics of scale might alter this picture. One such example is the recent announcement of Tesla, a producer of electric cars, to build a factory for stationary battery storage systems that are to be sold at an unprecedented low price (Tesla 2015).

**Fig. 5:**
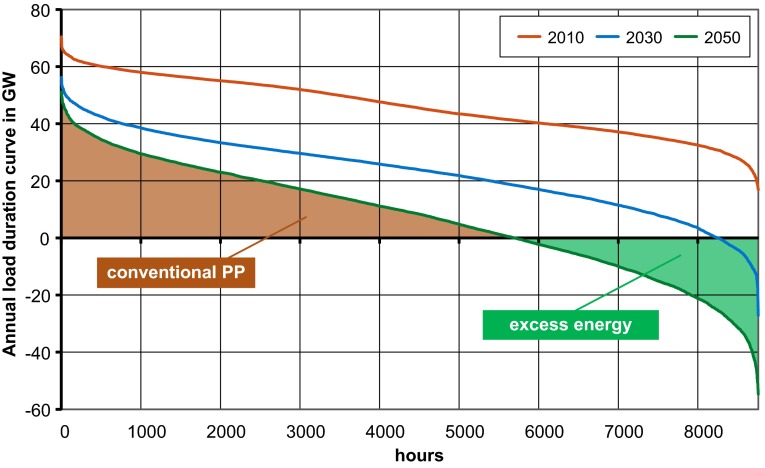
Sorted annual load duration curve—estimation for Germany until 2050

A first estimate of storage options can be derived from the sorted annual residual load curve. Supply of intermittent renewable sources is subtracted from the hourly demand and the remaining values are sorted by descending sequence. These curves show that even in a system with 80 % of wind and solar energy supply, many hours remain in which conventional power plants must supply the load (Fig. 5). Furthermore, we can see that generation exceeds demand in almost 3 000 h of the year 2050.

### Short literature review

Several studies (DLR 2010; UBA 2010; VDE-ETG 2012) analyze the future perspectives for storage technologies in Germany. They try to quantify the storage demand in the German electricity system until 2050, whereas the assumptions with respect to the development of load and renewables differ in a small range. Despite identical starting conditions, the results differ significantly because of varying models and input data. Therefore, the results are difficult to compare and a reasonable interpretation requires closer knowledge of all details, implying need for own research.

### Own research: Model/scenario description

**Fig. 6:**
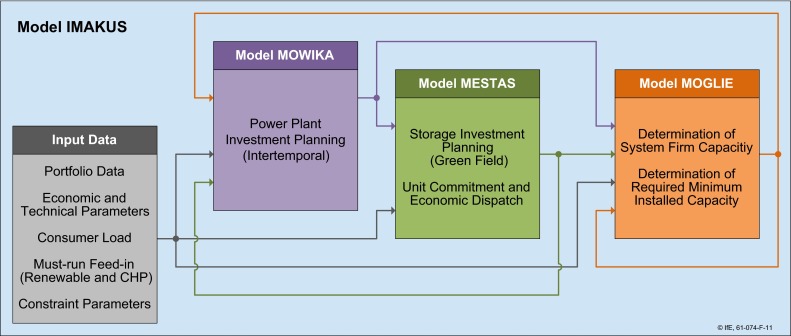
Overview of the IMAKUS-Model environment

The following results are taken from Kuhn et al. (2012). The goal of this work was to identify the storage demand in Germany in the coming decades if the renewable (wind, solar) sources go up to 80 % of the supply in the year 2050. The special modeling environment IMAKUS was designed to mix an inter-temporal generation expansion planning with an annual storage optimization model which could well capture the nature of intermittent supply sources (Fig. 6). The complete model can estimate the storage demand for the next decades and the optimal conventional power plant mix to back up the renewable sources under the constraint of a renewable supply mix.

### Own research: Findings

**Fig. 7:**
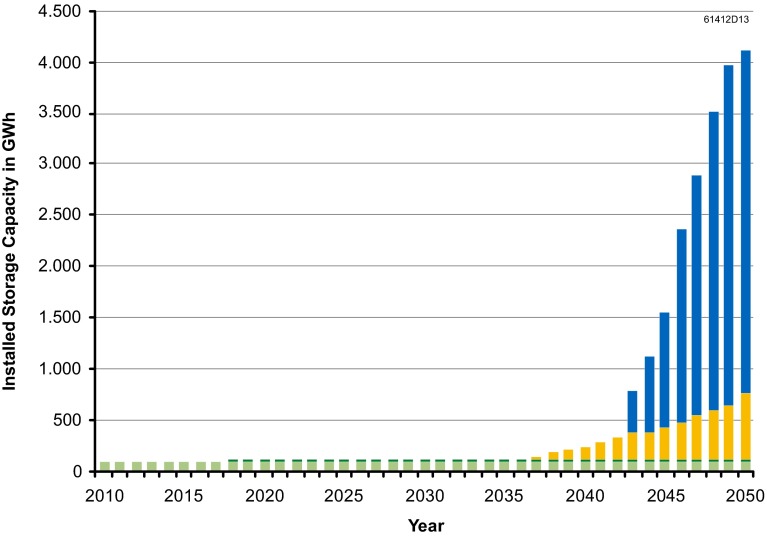
Storage capacity increases dramatically in future (Kuhn et al. 2012). *Green* PSH, *yellow* AA-CAES, *blue* hydrogen

Similar to the studies mentioned above, the results strongly depend on input parameters and small variations can lead to considerable changes in the results. Still we can derive some very important qualitative characteristics which are necessary to understand the future research demand. First, we see that the storage potential is enormous on the long term, with capacities being one or two orders of magnitudes higher than that of existing pumped storage (Fig. 7). The current technology—more or less only pumped hydro storage (PSH)—is certainly not feasible to integrate high shares of intermittent renewable electricity generation due to topography and environment. New technologies need to be developed. The two alternatives in the model are advanced adiabatic compressed air energy storage (AA-CAES) and hydrogen. Compressed air is only employed by the model because of constraints on the further use of pumped storage options. Hydrogen in contrast is also applied independent from a limit on pumped storage capacities. This can be well explained by their different cost structures for storage power (€/MW) and capacity (€/MWh). The major advantage of hydrogen is its higher energy density, leading to lower costs for storage capacity. This holds more or less for all chemical storage options.

### Future: Chances and regulatory problems

The study shows that on the diurnal and seasonal time scales, new storage options need to be developed. The seasonal storage options require a conversion of electricity to chemical energy. In a very simplified view, the process could be divided in two steps: the production of hydrogen from electricity and the refinement of hydrogen to a storable commodity. Both process steps need technical and economic improvements in order to be extended to large-scale applications.

Pumped hydro storage can provide diurnal storage but the potential is limited. Alternatives are either compressed air plants like AA-CAES and Electro Thermal Energy Storage (ETES), or decentralized options like batteries or super-capacitors. For AA-CAES, high-temperature compressors and high-temperature heat storage options need to be developed. Certainly, breakthroughs are expected here in the coming years.

Despite these technological challenges, national governments have regulatory power to both foster more research and change regulations in a way that puts a higher value on storage in the power system.

## International solution

### Motivation/intended effect

Finally, we are widening the scope to transnational concepts of future electricity systems. The interchange between countries requires large transmission capabilities that can be provided by setting up a so-called super-grid. The term super-grid describes mostly a power grid spanning over a whole continent. In most cases, another layer, mostly designed as high-voltage DC grid, complements the “conventional” high-voltage AC grid.

**Fig. 8:**
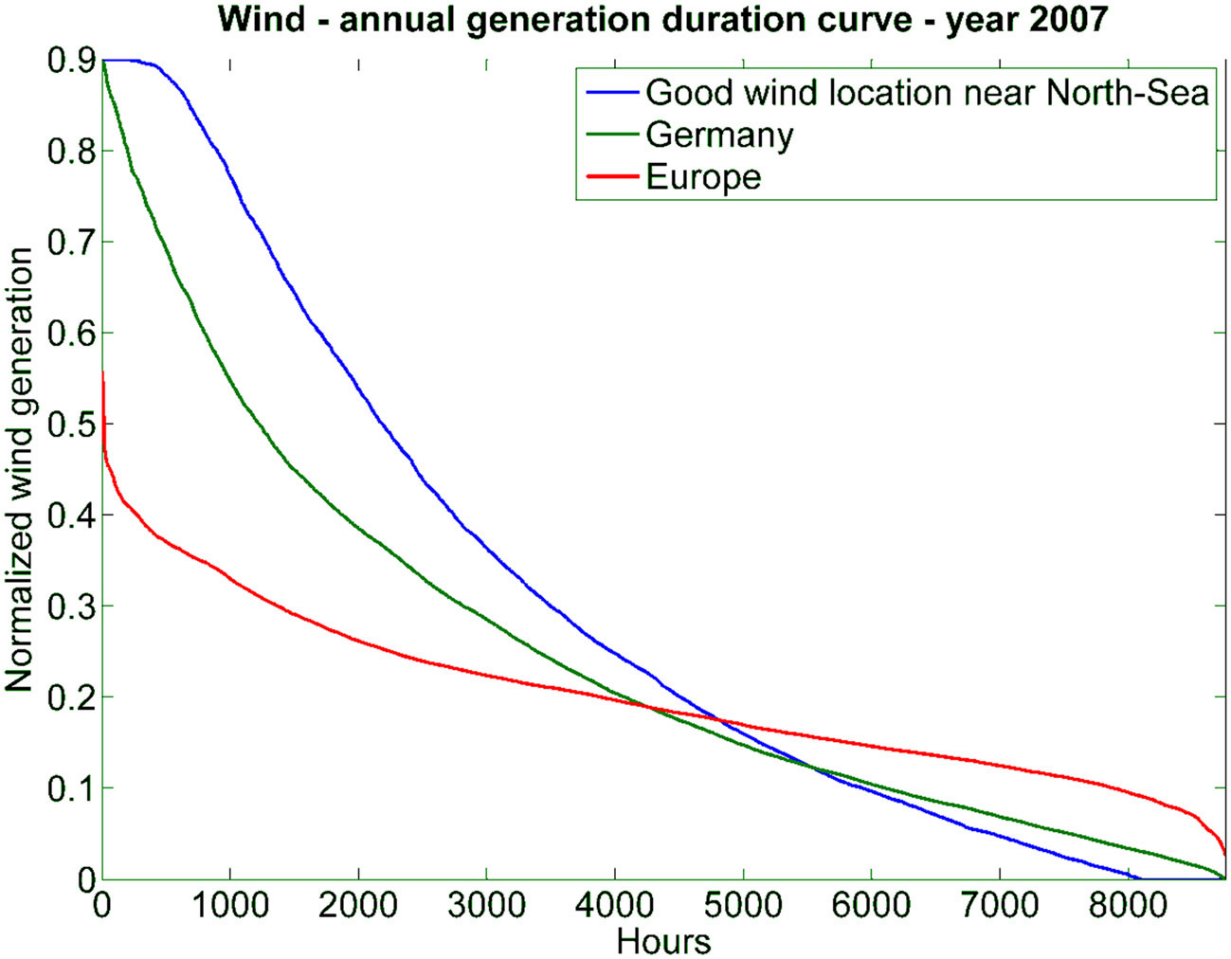
Sorted supply curves of wind power for three different regional allocations

The rationale behind this idea is twofold. First, many sites for wind and solar installations are far away from demand centers, and second, the summation of many intermittent sources leads to a smoothing effects. The latter fact can be easily demonstrated analyzing wind data. Figure 8 shows the wind generation for three different cases: (1) the generation of an individual wind turbine at a very good site, (2) the sum of many wind turbines dispersed over the whole of Germany, and (3) the sum of wind generation of turbines distributed in the whole of Europe. Wind would supply at least a small fraction of power at any time only in the European case and also the variation of supplied power is much smaller for the European case.

Historically, energy policy has already been a strong driver of forming the EU. Forming the European Coal and Steel Community (ECSC) in 1951 and EURATOM as part of the Roman Treatise in 1957 marked the beginning of the process European integration. In the recent past, the liberalization of the electricity market in 1998 and the gas market in 2000 as well as the introduction of the European Union Emission Trading System (ETS) needs to be mentioned.

### Short literature review

Research on continent-wide electricity systems has already been conducted extensively. Concerning analysis of generation patterns, research was done that investigates optimal mixes of wind and solar generation for the whole European continent. Heide et al. (2010) found an optimal mix of 55 % wind and 45 % solar generation for Europe based on minimizing excess electricity. Schaber et al. (2012a, b) found that the optimal mix depends on the grid extension: With better grid integration, the optimal share of PV is lower. For a system with 60 % renewable and a fully extended grid, the optimal share of PV is found to be only 15 %.

Grid extensions play a very important role when aiming at a European power system with high shares of renewables. Schaber et al. (2012a) found that backup capacity requirements and overproduction can be reduced significantly when setting up large transmission capacities. In Schaber et al. (2012b), the same authors emphasize the importance of grid extensions for different regions across Europe. Grid extensions will help the backup capacities to be economically viable and thus help incentivize investments. Becker et al. (2014) added the inter-temporal dimensions and analyzed the system development until the year 2050. The results confirm the positive effects of transmission grids for even longer time horizons. Rodríguez et al. (2014) investigated scenarios with exactly 100 % supply of wind and solar and find that a transmission grid that has 11.5 times higher capacities for inter-country connections allows to earn the complete benefits of international cooperation. Steinke et al. (2013) also investigated 100 % renewable scenarios and the results confirm the line of arguments even when storage options are considered.

Advantages of a European super-grid go beyond reducing overproduction and reducing backup capacities. Huber et al. (2014) analyze different mixes of wind and solar with regard to required flexibility for balancing the residual loads. The authors thereby found additional benefits from large transmission lines that lie in reduced ramps and, therefore, an easier balancing of generation and load.

One step beyond, research on even larger systems was conducted. Czisch (2005) investigated the chances that super-grids offer for an easier integration of large-scale renewable energies. He found that a fully renewable system is possible at moderate costs when Europe and North Africa are connected. Aboumahboub et al. (2010) as well as Chatzivasileiadis et al. (2013) investigated scenarios with a super-grid that not only spans across a continent but across the whole world.

All those studies found benefits from international cooperation in Europe and even globally. Our own research presented here focuses on the benefits for Europe as well but additionally considers generation possibilities in North Africa and Middle East and therewith extends the previously mentioned studies.

### Own research: Model/scenario description

In order to estimate the positive effects of this transcontinental power system, the model framework URBS is applied to find a cost-optimal infrastructure allocation for the electricity supply in the EUMENA region (European Union, Middle East, and North Africa).

**Fig. 9:**
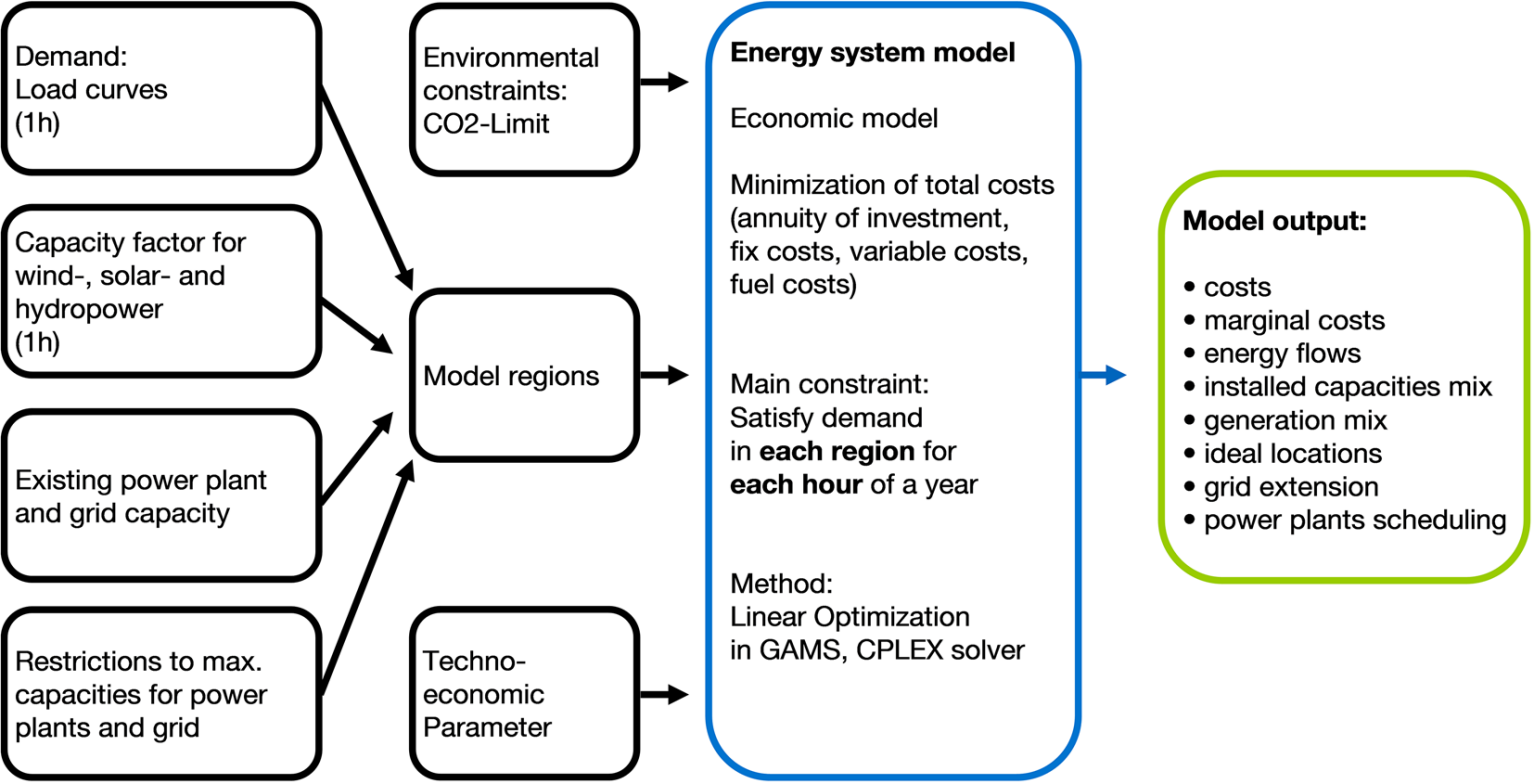
Overview of URBS modeling framework

The model URBS was originally developed by Richter (2004) and was applied in several research projects on optimal planning of the future electricity system. The model works on a time resolution of 1 h and a spatial resolution of countries. The model finds a cost-optimal extension of generation, transmission, and storage while considering an hour-by-hour dispatch. An overview of the model input and output is illustrated in Fig. 9 and a complete description of the model can be found in Huber et al. (2012).

**Fig. 10:**
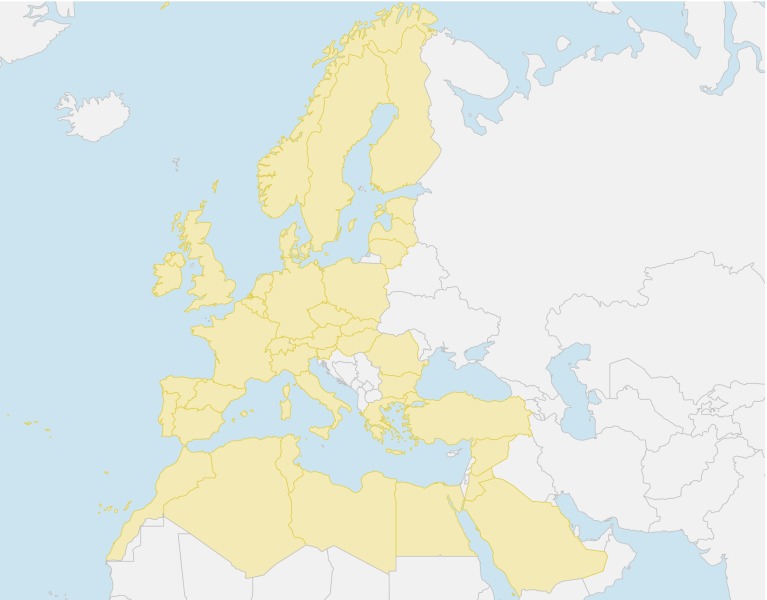
Map of investigated EUMENA countries. Data: Natural Earth

In this case study, the EUMENA region is represented by 39 regions (Fig. 10). Data for each region include hourly demand, hourly feed-in of renewables, and specific data for already existing infrastructure. As a major assumption, the maximal CO_2_ emissions for the whole EUMENA region are restricted to be less than 95 % of the 1990 emissions. The detailed description of all input parameters that also included price assumptions for fuels and facilities is given in Huber et al. (2012).

### Own research: Findings

**Fig. 11:**
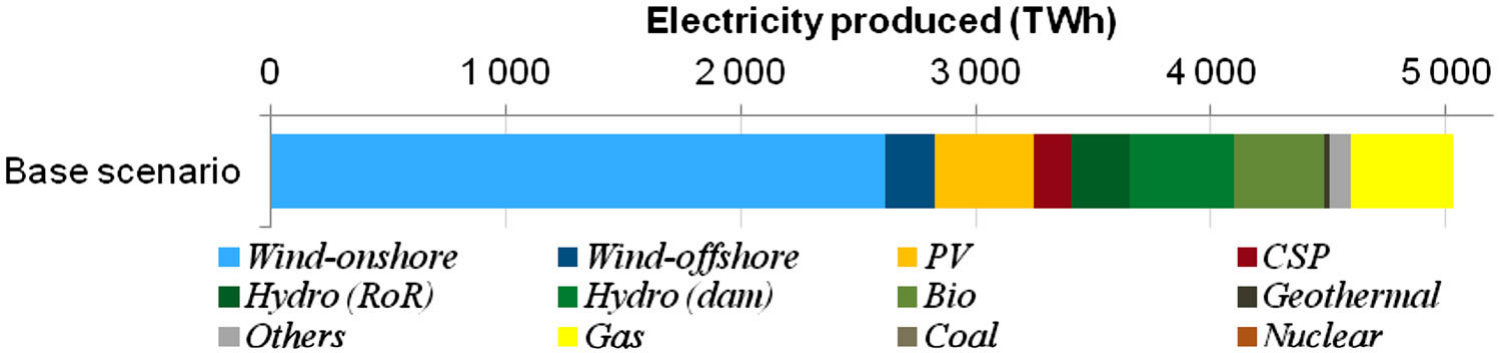
Cost-optimal electricity generation in a low-carbon electricity system of the EUMENA region (Huber et al. 2012)

The results of this study allow us to draw several important solutions regarding future design of the electricity system and respective research needs. In Fig. 11, the generation for the whole EUMENA region in a cost-optimal setting is displayed. We can draw several observations and conclusions from the results. First, it can be easily stated that in most scenarios wind energy plays a major role. Wind supplies up to 50 % of the overall electricity production in most scenarios. This fact is explained on one hand by the low costs of electricity for very good wind sites and on the other hand by the above-mentioned smoothing effects for wind generation. The result is rather robust under variations of input parameters that were considered in scenarios. One scenario included the chances for a nuclear technology, which can be either fission or fusion, and finds that a large transmission grid would allow to have a system of renewables combined with such base load plants (Hamacher et al. 2013).

**Fig. 12:**
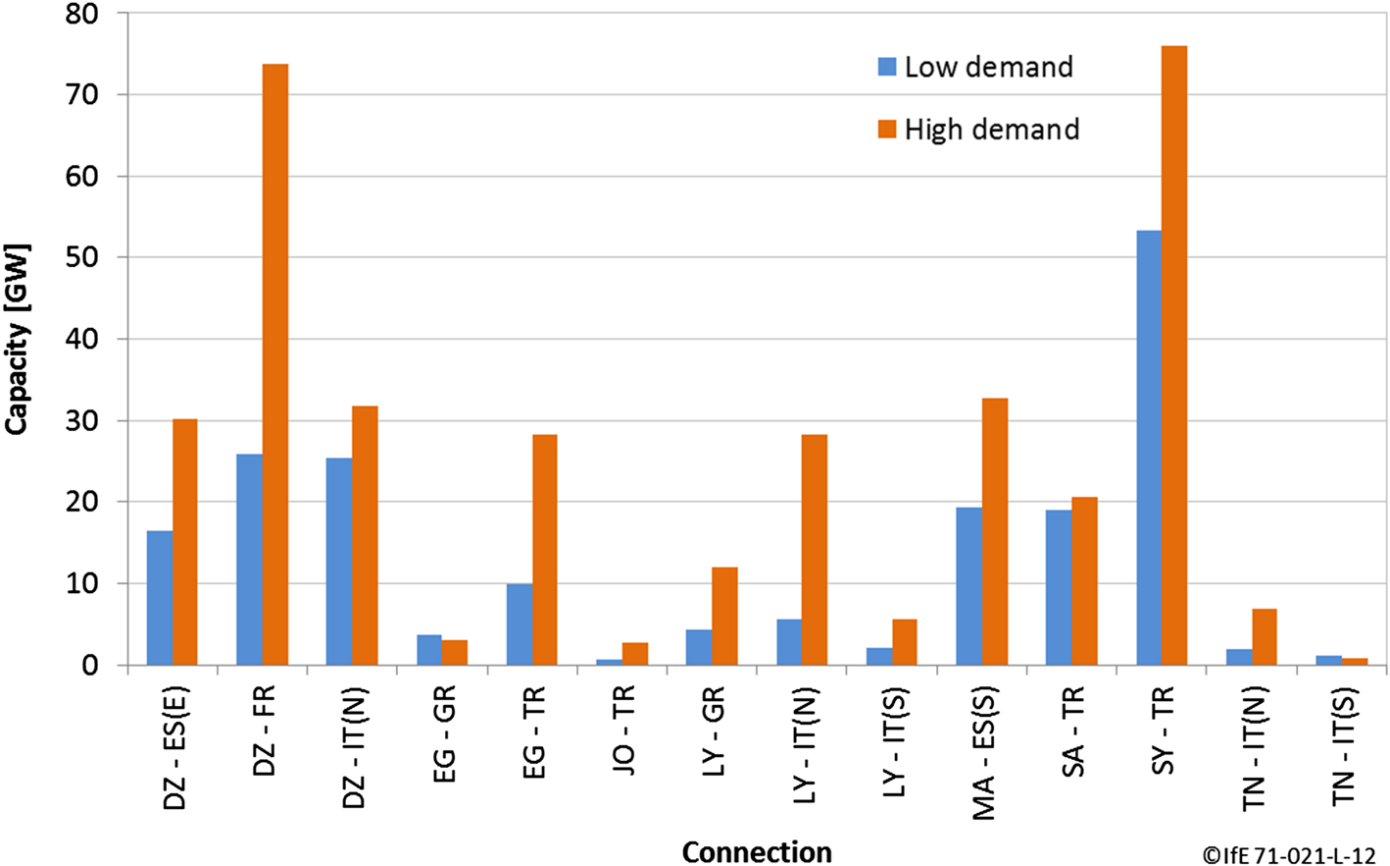
International transport capacities for a cost-optimal electricity system in the EUMENA region (Huber et al. 2012)

The optimal power grid extensions seem unreachable even under optimistic projections of the technical and political constraints: Fig. 12 shows that transmission capacities between countries should be as high as 70 GW for the high-demand scenario between Algeria and France. For comparison, today’s largest cross-border connection in the European ENTSO-E power grid has a capacity of 4.1 GW between Italy and Switzerland (ENTSO-E 2015). Even for the low-demand scenario, very high transmission would be required, the highest value being 50 GW of transmission between Syria and Turkey. Still, the direction of the results and the literature is clear: transmission grid extensions are a highly effective measure for large-scale renewable integration.

### Future: Opportunities and regulatory problems

The literature review as well as own research clearly showed positive effects of setting up large transmission capacities. Such systems would require cost-effective and efficient transmission technology that can be high-voltage DC lines. Research should therefore be undertaken in this direction. We also showed that wind will be the favored technology in the scenarios and should be a focus or research and development as well. Huge storage capacities are not required when aiming at those systems, as balancing occurs from interregional differences of generation patterns. This fact also allows for large centralized base load plants that could be nuclear fusion in the far future.

Large-scale transmission grid extensions, enabled by mutual dependency between countries could be a very promising approach. Maybe it is even the best option from a techno-economical perspective, but its limitations arise from socio-political constraints. At this moment, even the European Union faces challenges to uphold trust among members. Energy is essential for the functioning and competiveness of countries and thus pushes nationalism in many member states. Still, stronger cooperation could have many benefits for all participating countries. Especially countries that face severe economic challenges could profit. Creutzig et al. (2014) already discussed similar scenarios in detail and found energy cooperation to be a beneficial stimulus program for struggling EU members. Southern countries like Greece, Italy, Spain, and Portugal have the highest unemployment rates but very good sites for renewable generation and electricity export. Altogether, it can be said that international cooperation has many potential benefits but political obstacles. Research and policies should try to solve those issues and establish a profitable energy cooperation.

Until now, the European cooperation is still challenged by national peculiarities, while the ETS suffered from a decay of prices making it a toothless tiger. If these challenges can be overcome, the European solution offers a long-term low-cost path, which should encourage European bodies to strengthen a unified European energy policy including greenhouse gas emission reduction, promotion of renewables, and a common infrastructure build-up. A new energy policy pushing for renewable energy sources and emission reduction could be a major European project of the future which might at some point turn out to be as important as the ECSC was more than 50 years ago.

## Summary

This paper has presented potential solutions to the challenges of future power systems. Depending on the scope of the investigated system, three substantially different “optimal” solutions emerged: First, a local solution on the scale of districts or cities would require substantial investment in technical flexibility options (especially storage). Even with these options in place, only partial independence from a transmission grid could be achieved. Although absolute autonomy would in principle be achievable, the sheer price for the required technical assets will limit such solutions to very few cases. An interesting concept is microgrids that enable decentralized energy management. Second, the national solution aims to balance renewable electricity production and consumption on a per-country level. The national approach already reduces the required short-term flexibility due to smoothing of demand and intermittent energy supply patterns. However, seasonal balancing still is an enormous technical challenge ahead. An interesting option is coupling the power and heat sectors. Finally, the international solution builds on installing a large-scale super-grid that spans at least one continent. The scaling effects at this size promise to finally bring technical challenges down to a magnitude that could be met by moderate investments in already proven technology.

**Fig. 13:**
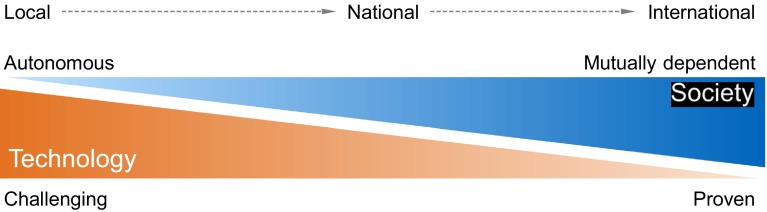
Trade-off between social and technological complexity, depending on scope of measures

The broad properties of the three solutions are summarized in Fig. 13. Only time will tell which scope(s) will become more prevalent. From a techno-economic standpoint, solutions that build on cooperation tend to be more favorable. But only political stability can ensure that such a cooperation really leads to benefits for all involved partners.
